# Nanostructured Lipid Carriers for Enhanced Transscleral Delivery of Dexamethasone Acetate: Development, Ex Vivo Characterization and Multiphoton Microscopy Studies

**DOI:** 10.3390/pharmaceutics15020407

**Published:** 2023-01-25

**Authors:** Felipe M. González-Fernández, Andrea Delledonne, Sara Nicoli, Paolo Gasco, Cristina Padula, Patrizia Santi, Cristina Sissa, Silvia Pescina

**Affiliations:** 1 ADDRes Lab, Department of Food and Drug, University of Parma, Parco Area delle Scienze, 27/a, 43124 Parma, Italy; 2 Nanovector S.r.l., Via Livorno, 60, 10144 Torino, Italy; 3 Department of Chemistry, Life Science and Environmental Sustainability, University of Parma, Parco Area delle Scienze 17/A, 43124 Parma, Italy

**Keywords:** NLC, dexamethasone acetate, dexamethasone, posterior segment, ex vivo, ocular delivery, tyloxapol, multiphoton microscopy

## Abstract

Corticosteroids, although highly effective for the treatment of both anterior and posterior ocular segment inflammation, still nowadays struggle for effective drug delivery due to their poor solubilization capabilities in water. This research work aims to develop nanostructured lipid carriers (NLC) intended for periocular administration of dexamethasone acetate to the posterior segment of the eye. Pre-formulation studies were initially performed to find solid and liquid lipid mixtures for dexamethasone acetate solubilization. Pseudoternary diagrams at 65 °C were constructed to select the best surfactant based on the macroscopic transparency and microscopic isotropy of the systems. The resulting NLC, obtained following an organic solvent-free methodology, was composed of triacetin, Imwitor® 491 (glycerol monostearate >90%) and tyloxapol with Z-average = 106.9 ± 1.2 nm, PDI = 0.104 ± 0.019 and zeta potential = −6.51 ± 0.575 mV. Ex vivo porcine sclera and choroid permeation studies revealed a considerable metabolism in the sclera of dexamethasone acetate into free dexamethasone, which demonstrated higher permeation capabilities across both tissues. In addition, the NLC behavior once applied onto the sclera was further studied by means of multiphoton microscopy by loading the NLC with the fluorescent probe Nile red.

## 1. Introduction

Corticosteroids, first used in ophthalmology in the 1950s, still have a fundamental role in the management of ocular inflammation [1]. Triamcinolone acetonide, fluocinolone acetonide and dexamethasone, among others, usually represent the first-line treatment of inflammation related to conjunctivitis, non-infectious uveitis, macular edema, age-related macular degeneration, and even diabetic retinopathy [2].

Dexamethasone (Dex), a potent glucocorticoid, not only downregulates the expression of multiple proinflammatory chemokines and cytokines, but also inhibits retinal neovascularization. Nonetheless, its high lipophilicity hinders effective therapeutic doses when applied topically and several approaches have been proposed over the years [3]. Water soluble derivatives, such as dexamethasone sodium phosphate, have been developed to obtain more concentrated eye drops, while more lipophilic prodrugs, such as dexamethasone acetate (DexAc) have been selected to promote drug permeation across corneal epithelium [4,5,6]. Furthermore, to effectively address the back of the eye, dexamethasone-loaded implants (Ozurdex®) are administered intravitreally [1]. 

Several examples of research studies involving dexamethasone-loaded nano- and micro-systems are available: from liposomes [7,8], micelles [9,10], and nanostructured lipid carriers (NLC) [11,12] to nanowafers [13,14] and microparticles [15]. On the contrary, only a few studies involve dexamethasone acetate, as in the case of poly(D,L-lactide-co-glycolide) nanoparticles intended for intravitreal injections [16,17], cationic nanoemulsions [18], nanofibers for intravitreal injections [19], and polymyxin B-coated NLC containing DexAc for treatment of infection-related eye surface inflammation [20]. These approaches, based on nano- or micro-systems, are believed to improve therapeutic success by (i) reducing the frequency of administration with the possibility of a prolonged and sustained release, (ii) lowering the invasiveness of administration routes (i.e., periocular administration routes), and (iii) limiting side effects by effective drug targeting at low dosage levels [21]. 

Among the different nanosystems, lipid-based carriers both solubilize and protect poorly water-soluble drugs within biocompatible lipid matrices mainly composed of generally recognized as safe (GRAS) lipids while improving bioavailability due to a high specific surface area [22,23,24]. Nanostructured lipid carriers (NLC) are second-generation lipid-based nanocarriers, consisting of a core matrix of solid and liquid lipids stabilized by a surfactant-cosurfactant mixture, with improved drug loading and stability in comparison to early solid lipid nanoparticles (SLN), i.e., nanoparticles composed of solid lipid cores surrounded by surfactants. The addition of a liquid lipid to the nanoparticle core avoids rearrangement into densely packed lattice structures, which has been identified as a limitation of SLN since it might lead to premature drug expulsion [25]. Effective retinal delivery of lipophilic agents, such as dexamethasone acetate, can benefit from this approach to overcome ocular barriers, thus achieving therapeutic doses [26].

In the present work, we aim to develop dexamethasone acetate-loaded nanostructured lipid carriers intended for periocular administration. Pre-formulation studies involving lipid polymorphism characterization and solubility assessment were performed to find the adequate ratio between lipid, surfactant and water that yields warm microemulsion templates for NLC production. A straightforward methodology that does not require the use of organic solvents, whose remnants of the production process could be toxic to the eye, was followed and the obtained NLC were characterized for their permeation and accumulation capabilities on ex vivo swine ocular tissues. Loading of the NLC with the fluorescent probe Nile red (NR), permitted high-definition images by multiphoton microscopy, elucidating the behavior of the NLC once applied on excised porcine sclera. 

## 2. Materials and Methods

### 2.1. Materials

Dexamethasone acetate (DexAc; IUPAC [2-[(8S,9R,10S,11S,13S,14S,16R,17R)-9-fluoro-11,17-dihydroxy-10,13,16-trimethyl-3-oxo-6,7,8,11,12,14,15,16-octahydrocyclopenta[a]phenanthren-17-yl]-2-oxoethyl] acetate; MW 434.5 g/mol; LogP 2.65; water solubility 10 µg/ml) [27] was purchased from TCI Europe N.V. (Zwijndrecht, Belgium).

Dexamethasone (Dex; IUPAC (8S,9R,10S,11S,13S,14S,16R,17R)-9-fluoro-11,17-dihydroxy-17-(2-hydroxyacetyl)-10,13,16-trimethyl-6,7,8,11,12,14,15,16-octahydrocyclopenta[a]phenanthren-3-one; MW 392.5 g/mol; LogP 1.9; water solubility 89 µg/ml) [28] and tyloxapol (IUPAC 4-(2,4,4-trimethylpentan-2-yl)phenol; formaldehyde; oxirane) were from Sigma Aldrich (Taufkirchen, Germany). Triacetin (glycerol triacetate, colorless liquid; melting temperature 3 °C) and Nile red (NR; IUPAC 9-(diethylamino)benzo[a]phenoxazin-5-one; MW 318.37 g/mol) were purchased from TCI Europe N.V. (Zwijndrecht, Belgium).

Imwitor® 491 (glycerol monostearate, GMS) (Rahway, NJ, USA), off-white powder; ≥90% content in monostearate; melting temperature 66–77 °C) was kindly gifted by IOI Oleo GmbH (Hamburg, Germany). The vitamin E-derived surfactant tocopherol polyethylene glycolsuccinate (TPGS; MW approx. 1513 g/mol) was a kind gift of PMC ISOCHEM (Vert-Le-Petit, France).

Saline solution (composition: 9 g/L NaCl) and phosphate buffered saline (PBS; composition: 0.19 g/L KH_2_PO_4_, 2.37 g/L Na_2_HPO_4_, 8.8 g/L NaCl; pH 7.4 by adding 85% H_3_PO_4_) were prepared using ultra-pure water (Arium^®^ Comfort Sartorius, Goettingen, Germany).

All other chemicals were of analytical grade.

### 2.2. Lipid Solubility Screening

A simple method for quick drug solubility screening in both liquid and solid lipid matrices compatible with the ocular route was developed, with modifications from [29]. Briefly, five milligrams of dexamethasone acetate was accurately weighed in transparent glass vials. Aliquots of the selected oils were added at room temperature under magnetic stirring until optical transparency was observed, allowing the system to stabilize for five minutes between each aliquot. For solid lipids, each sample was heated ~5 °C over the melting point and the same procedure was followed. The endpoint was considered to occur when no signs of turbidity were macroscopically observed. Results were confirmed in triplicate.

### 2.3. Oil-Solid Lipid Mixture Characterization by Differential Scanning Calorimetry

Binary mixtures at different ratios of the selected oil and solid lipid were characterized by differential scanning calorimetry (STAR-e System DSC 1, Mettler Toledo, Columbus, OH, USA). To this end, 11 ± 1 mg of material was accurately weighted in 100 µL aluminum crucibles and sealed. DSC apparatus was calibrated with indium calibration standard (for melting point and heat of fusion). Data were obtained following heating ramps from 25 to 90 °C at a 2 °C/min heating rate. Polymorphic events were recorded at 5 min, one day, one week and 1 month after solidification of the melts.

### 2.4. Surfactant Screening

Pseudoternary phase diagrams were constructed with three different ocular compatible surfactants using a water titration technique. Briefly, initial mixtures containing only lipid mix (80% GMS: 20% triacetin) and a surfactant (either Tween® 20, Tween® 80 or tyloxapol, (Rahway, NJ, USA)) were accurately weighted in varying ratios (ranging from 9:1 to 1:9 in weight) in transparent glass vials. The mixtures were heated at 65 °C under magnetic stirring. Aliquots of 10% (with respect of the initial lipid mix/surfactant weight) of ultra-pure deionized water were progressively added and the macroscopic appearance after stabilization was noted. The lipid mix/surfactant/water mixtures were classified as either macroscopically transparent or not, with no effort to further characterize the non-transparent formulations. Data were plotted using OriginPro^®^ 2019 (OriginLab, Northampton, MA, USA).

### 2.5. Hot-Stage Polarized Light Microscopy

An optical polarizing light microscope (Nikon Optiphot 2 POL, Nikon, Melville, NY, USA) coupled to a stage temperature microscope controller (TP 93 Linkam Scientific Inst., Miami, FL, USA) allowed for microscopic confirmation of isotropy of the selected candidates of the pseudoternary system lipid mix (60% GMS: 40% triacetin)/tyloxapol/water. A drop of warm sample was deposited on a microscope slide and a cover glass was carefully placed. Samples were positioned on the hot stage, preheated at 65 °C, and observed under cross-polarized light immediately, minimizing any possible evaporation. Photomicrographs were taken at 400× magnification.

### 2.6. NLC Preparation and Characterization

A modified method based on previously published work by Cavalli and colleagues [30], with some modifications from [31] was followed. Briefly, the lipid mix (60% GMS: 40% triacetin), surfactant (tyloxapol), and drug (DexAc) were accurately weighted in an 8 mL glass vial with a screw cap and heated under magnetic stirring to 65 °C. After stabilization of the system, 2.25 mL of preheated ultra-pure deionized water was added at the same temperature and the system was stabilized for 5 min. The warm microemulsion was dispersed 1:20 in 47.5 mL of cold water (~2 °C) with a sonicating probe at a constant frequency of 30 kHz (amplitude 100% (180 µm), Cycle 1; UP100H ultrasonic processor, Hielscher ultrasonics, Teltow, Germany). The dispersed NLC was placed for 10 min at −20 °C, followed by 10 min in a sonicating bath (Branson 2510 Ultrasonic Bath Sonicator, Branson Ultrasonics, Danbury, CN, USA).

After 24 h, the obtained NLC was washed from unencapsulated drug and concentrated by means of a tangential flow cartridge, following manufacturer instructions (Vivaflow® 100 kDa, Sartorius, Göttingen, Germany). Samples were characterized for size and zeta potential, and drug loading was determined after digestion of NLC with tetrahydrofuran (1:5, *v*/*v*) at room temperature for 15 min. Aliquots of the digest were diluted 1:10 with an extraction mixture of acetonitrile and water in ratio 65:35 (*v*/*v*) and submitted for HPLC-UV analysis.

The same procedure was followed for the preparation of Dex-NLC.

#### 2.6.1. Dynamic Light Scattering (DLS)

A Malvern Zetasizer Nano ZS (Malvern Panalytical Ltd., Malvern, UK) was used for nanoparticle size and zeta potential measurement. The measurements were performed at 25 °C, with an incidence angle of 173° (refractive index 1.33; viscosity 0.8872 cP). For the size measurement, samples were diluted 1 to 50 with ultra-pure deionized water. Size distribution was reported as Z-average obtained from intensity distribution. For zeta potential characterization, samples were diluted in a 10 mM NaCl solution and measured in a zeta potential cell.

#### 2.6.2. Nile Red-Loaded NLC

Nile red-loaded NLC (NR-NLC) was produced following the aforementioned methodology, substituting pure triacetin with a 0.1 mg/mg or 0.5 mg/mg solution of Nile red in triacetin.

Unencapsulated Nile red was separated by size exclusion chromatography (PD-10 Desalting Columns, Sephadex™ G-25 M, Cytiva, UK). One milliliter of loaded NLC was eluted through the column by stepwise addition of 1 mL volume of ultra-pure water. A total of eight fractions were collected. Fractions 4 and 5 appeared colored to the naked eye and were then analyzed by DLS for confirmation of NLC presence. Separation efficiency was further checked via fluorescence measurements, which also provided spectroscopic properties of fluorescent-NLC (see Section 2.6.3).

#### 2.6.3. Spectroscopy Studies

Both blank and NR-loaded NLC were spectroscopically characterized to investigate Nile red encapsulation. UV-Vis absorption spectra were acquired with a PerkinElmer Lambda650 spectrophotometer (PerkinElmer, Waltham, MA, USA), while a FLS1000 Edinburgh fluorometer was used to perform fluorescence measurements in solution and aqueous suspension. Fluorescence spectra were collected on diluted samples, with absorbance lower than 0.1 (to avoid inner filter effects) and further corrected for the excitation intensity and detector sensitivity. Absorption spectra of the NLC aqueous suspensions were inevitably convoluted to the scattering signal, which dominated most of the absorption profile. Appropriate longpass filters have been inserted in the emission path to collect fluorescence spectra of NLC suspensions, to minimize the scattering of the excitation light.

### 2.7. Ex Vivo Swine Ocular Tissues

Swine ocular bulbs (*Sus scrofa domestica)* were obtained from a local abattoir (Macello Annoni S.p.a., Busseto, Italy) and transported under refrigeration in saline solution and manipulated within 4 h upon arrival. Ocular adnexa were carefully removed with surgical Mayo scissors and the intact ocular bulbs were opened by means of a perilimbal incision with a scalpel. Anterior ocular sections, and vitreous body were removed. Retina and retinal pigmented epithelium (RPE) were thoroughly removed with the help of filter paper. The ocular bulb was halved following the line of the ciliary arteries and the half without optic nerve, consisting of sclera with attached choroid or plain sclera, was used.

### 2.8. Validation of an DexAc-Dex Extraction Method from Porcine Sclera and Choroid

Circular sections (9 mm diameter) of fresh and thawed (previously stored at −20 °C) sclera and choroid were punched and accurately weighted in 1.5 mL Eppendorf® tubes. Then 2.5 mg/mL stock solutions of both DexAc and Dex in ethanol 90% were prepared and 10 µL was carefully applied onto the isolated tissues, allowing for solvent evaporation. After 30 min, an extracting mixture of acetonitrile and water (65:35, *v*/*v*) was added (1 mL for the sclera and 0.5 mL for the choroid). Tissues were left in contact with the extracting mixture for 2 h at room temperature with eventual sample vortexing. Supernatants were taken for HPLC drug quantification.

### 2.9. Permeation and Retention Experiments

Isolated sclera or sclera with attached choroid were mounted on Franz-type vertical glass diffusion cells with a 0.6 cm^2^ permeation area. The donor contained 200 µL of the formulations of interest with DexAc concentrations ranging from 200 to 240 µg/mL (corresponding to 460.3–552.4 μM). As a reference, a 250 μg/mL DexAc solution in triacetin and a 200 µg/mL Dex-NLC were used. The receptor chamber contained a defined volume (approx. 4 mL) of a 0.5 mM TPGS solution in PBS (pH 7.4), previously degassed. Next, 300 µL aliquots were taken at predefined times of 0, 2, 4, 6, 20, 22 and 24 h, and replaced each time with fresh medium. After 24 h, the formulation was removed, and the scleral surface was accurately cleaned. Circular sections of 9 mm diameter, consisting of sclera and/or choroid corresponding to the contact area, were cut, weighted, and placed in extractive mixture for 2 h. Drug-free NLC and plain triacetin were used as blanks to exclude possible analytical interferences, while Dex-NLC was tested for comparison purposes.

### 2.10. Multiphoton Microscopy Studies

NR-loaded NLC was put in contact with fresh porcine sclera for 2 h using the Franz cell setup. After NLC removal and tissue washing, the sclera was punched to obtain 9 mm diameter discs. The distribution of NR within scleral tissue was studied via multiphoton microscopy (MPM). Scleral discs were placed into a customized plexiglass holder and moistened with saline solution to avoid dehydration and to maintain the contact between the objective of the microscope and the tissue. Equipment consisted of a two-photon microscope Nikon A1R MP+ Upright and a femtosecond pulsed laser Coherent Chameleon Discovery (~100 fs pulse duration with 80 MHz repetition rate, tunable excitation range 660–1320 nm). The excitation beam was focused on the sample by a 25× water dipping objective with numerical aperture (NA) 1.1 and 2 mm working distance. The two-photon excited fluorescence (TPEF) and the second harmonic generation (SHG) signals were collected by the same objective and directed by a dichroic mirror to two non-descanned detectors (high sensitivity GaAsP photomultiplier tubes) allowing fast image acquisition. The detectors are preceded by optical filters allowing the simultaneous acquisition of two separated channels: a green channel (506–593 nm) and a red channel (604–679 nm). Imaging overlay of the two channels and image processing was performed by the operation software of the microscope. Images were acquired with 1024 × 1024 pixels definition using 1100 nm as excitation wavelength. A third photomultiplier GaAsP detector, connected to the microscope through an optical fiber and preceded by a dispersive element, was used to record the spectral profile of the TPEF/SHG signal (from 430 to 650 nm with a bandwidth of 10 nm). Laser power and detector gains have been adjusted for different samples and different depths, to acquire enough signal for images and spectra.

### 2.11. HPLC Analysis

A HPLC/UV-Vis method allowing for simultaneous quantification of dexamethasone and dexamethasone acetate was developed, starting from [9]. Analysis was conducted with an Agilent 1260 Infinity apparatus (Agilent Technologies, Santa Clara, CA, USA). Separation occurred in a reverse phase column Nova-Pak® C18 (4 μm, 3.9 × 150 mm; Waters, Milford, MA, USA) heated at 40 °C. A mobile phase consisting of an acetonitrile and water mixture in ratio 45:55 (*v*/*v*) was pumped at 1 mL/min and detection was performed at 246 nm. Retention times of 2.1 min for dexamethasone and 5 min for dexamethasone acetate were observed.

Two calibration curves were constructed: one for extraction samples in organic extracting mixture (acetonitrile/water, 65:35, *v*/*v*) and one for permeation samples in aqueous phase containing 0.5 mM TPGS in PBS pH 7.4. Both dexamethasone and dexamethasone acetate expressed linearity in the 0.2–10 µg/mL concentration range when present in organic extracting mixture. The limit of quantification (LOQ) was 0.3 µg/mL when the drugs were present in the aqueous phase. For both drugs and calibration curves, limits of detection (LOD) were ≥ 0.1 µg/mL, precision (expressed as relative standard deviation percentage) was lower than 2.5% for all studied concentrations while the relative error, indicative of method accuracy, remained in any case under 10%.

### 2.12. Data Processing

The amount of Dex and DexAc permeated across sclera and sclera-choroid (nmol/cm^2^) was plotted against time (hour): the slope of the regression line at steady state represents the trans-scleral flux *J_SS_* (nmol/cm^2^ h). The apparent permeability coefficient *P_app_* (cm/s) was then calculated from Equation (1):

(1) $$P_{app}=J_{SS}/C_{d}$$

where *C_d_* (µM) is the concentration of the donor solution. In addition, the lag-time (h) was calculated as the intercept of the regression line at steady state on the x-axis.

### 2.13. Statistical Analysis

Data were reported as mean ± standard deviation, unless otherwise stated. The differences between values were assessed using Student’s t test and considered statistically significant when *p* < 0.05.

## 3. Results and Discussion

The use of lipid-based nanocarriers to address the challenging delivery to the posterior segment of the eye has shown increasing interest in recent years [24]. On this basis, NLC was selected for efficient delivery of dexamethasone, a potent corticosteroid, under its more lipophilic prodrug ester, dexamethasone acetate, to increase its affinity to lipophilic tissues [32].

Preformulation studies for selecting adequate excipients are hereby presented. First, we selected the solid and liquid lipids that better solubilize DexAc. We studied the polymorphic changes in solid lipid GMS crystallization due to addition of the liquid triacetin. An adequate surfactant, tyloxapol, was selected on the capabilities of rendering macroscopically transparent and isotropic microemulsions at 65 °C, which can serve as templates for NLC production once redispersed 1:20 in cold water. The ratio between lipid mixture, surfactant and water that yields adequate NLC with physicochemical characteristics suitable for the ophthalmic route was identified. The formulation candidate was further characterized on its ability to enhance permeation and retention of DexAc in ex vivo porcine sclera and choroid. Additional information about the NLC behavior when in contact with sclera was collected by multiphoton microscopy.

### 3.1. Lipid Solubility Screening

**Figure 1:**
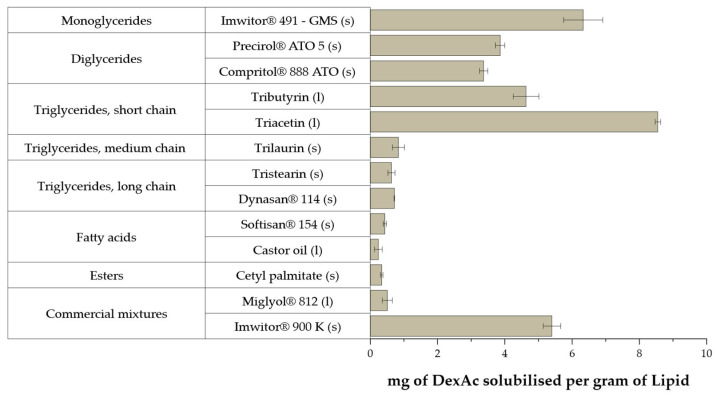
Solubility of dexamethasone acetate in diverse lipid matrices, liquid (l) or solid (s) at room temperature. Mean values are displayed ± standard deviation (*n* = 3).

DexAc solubility was measured in different lipid excipients, selected based on their ocular compatibility [33,34]. A total of 13 lipid matrices were evaluated and the results are presented in Figure 1.

The evaluated monoglycerides and diglycerides showed good solubilizing capabilities while, for the triglycerides series, only the short chain demonstrated adequate results. In fact, within the liquid candidates, exclusively triacetin could be selected since Miglyol® 812 and castor oil performed poorly. Within the solid fats, the best outcomes were observed for C18 diglyceride (Precirol® ATO 5) or monoglyceride (Imwitor® 491 and 900 K). Particularly, Imwitor® 491 is the glycerol ester of stearic acid, thus having an amphiphilic nature. Imwitor® 491 has higher (>90%) monostearin content than Imwitor® 900 K, which only contains 40–55% of GMS [35,36,37]. Commercial mixtures rich in diglycerides such as Precirol® ATO 5 or Compritol® 888 ATO, demonstrated lower solubility capabilities than GMS. Neither long- nor medium-chain triglycerides revealed good solubilization capabilities while short chains triglycerides, namely triacetin, demonstrated good DexAc solubilization.

Therefore, the solid lipid (Imwitor® 491, i.e., GMS) and the liquid lipid triacetin were selected based on their individual solubility capabilities for DexAc. GMS could act synergistically with the selected surfactant, due to its amphiphilic nature, thus reducing the surfactant content for obtaining nanoparticles. Triacetin is a saturated short chain triglyceride with very limited examples in the literature of applications in the ophthalmic route, although preliminary data on ocular safety show adequate ocular tolerability [38].

As a preparation for the DSC study, we investigated the solubility improvement by combining GMS and triacetin in different ratios, as presented in Figure S1. Triacetin shows excellent solubilizing capabilities and with decreasing triacetin content, the solubility is also reduced. In any case, adding only a 10% triacetin to the GMS already provided almost double the solubilizing capabilities (Figure S1).

No final decision was taken until DSC data were made available.

### 3.2. Liquid–Solid Lipid Mixture Characterization by Differential Scanning Calorimetry

Classical SLN, composed of only pure solid lipid cores, have been reported to lead to premature drug expulsion, since densely packed polymorphs do not offer free spaces for drug molecule inclusion [39,40]. Unlike SLN, NLC combine long chain with short chain glycerides that not only offer inclusion sites for the drug, but also alter the crystallization behavior upon solidification. In fact, up to three different NLC types have been identified so far based on the polymorphism observed in the lipidic core. The imperfect-type NLC (Type I) is obtained by combining (spatially different) long-chain and short-chain glycerides which create imperfections in the matrix structure that can accommodate the drug. The amorphous type (Type II) is obtained by adequately mixing lipids that generate matrices that remain in the amorphous state for a prolonged time avoiding crystallization and thus premature drug expulsion. Multiple type NLC (Type III) is obtained when the lipidic core contains oil compartments (or droplets) where the drug can allocate [25,41,42].

The polymorphic behavior of different ratio mixtures of triacetin and GMS (from 50 to 90% GMS content) was studied at 5 minutes, one day, one week and one month after solidification of the melt when preserved at room temperature. The DSC thermograms are depicted in Figure S2. The observed results are in close agreement with previously reported data for pure GMS (see Supplementary Materials for an in-deep analysis) [43,44,45,46,47,48]. Addition of a short-chain triglyceride, such as triacetin, can hinder rearrangement into densely packed lattices, thus avoiding premature drug expulsion from the NLC [25,49]. In our case, addition of triacetin to GMS delayed complete progression of the solid lipid toward densely packed rearrangements. In fact, less-ordered polymorphs, with lower melting temperatures, still subsist in the lipid matrix after one month when triacetin content is ≥ 20% in the lipid mixture. Considering also the previously obtained data on DexAc solubility, a GMS mixture containing 20% triacetin was selected for further studies.

### 3.3. Surfactant Screening

Several methods have been proposed to produce NLC, among which cold redispersion of warm microemulsions is a straightforward methodology [50]. Oil-in-water (O/W) microemulsions are thermodynamically stable, self-assembled systems with a particulate size under 200 nm mainly composed of a lipid core stabilized by surfactant(s) in aqueous media. O/W microemulsions obtained with solid lipids at high temperatures (above the melting point of the lipid) have been proposed as templates to produce SLN and NLC by means of rapid solidification of the melted core by redispersion in cold media [51].

This production technique requires quick methodologies to easily identify the best ratios between water, lipid and surfactant that yield adequate warm O/W microemulsions. In this sense, a quick method for screening of surfactants based on water titration techniques, typically proposed for microemulsions at room temperature, was applied at 65 °C with similar results. The progressive addition of water to a lipid/surfactant mixture allows quick identification of the lipid/surfactant/water mixture that yields colloidal systems that appear transparent to the naked eye, allowing individualization of an initial region in the pseudoternary phase diagram of the component mixture, as a possible template for our NLC production.

**Figure 2:**
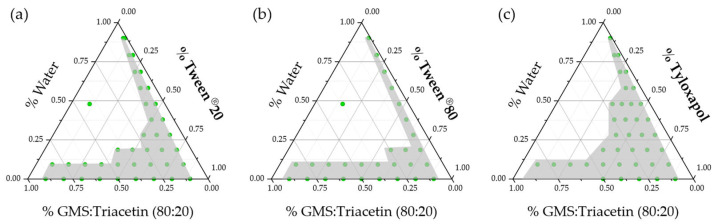
Pseudoternary phase diagrams for the selected lipid mix (80% GMS: 20% triacetin)/surfactant/water mixtures at 65 °C: (**a**) Tween® 20, (**b**) Tween® 80, and (**c**) tyloxapol. Green points represent the studied mixtures that yield macroscopically transparent systems defining an area (indicated in grey) of possible microemulsion templates for the NLC production.

Three ocular compatible non-ionic surfactants, namely Tween® 20, Tween® 80 and tyloxapol, were studied and the resulting pseudoternary phase diagrams are presented in Figure 2.

No differences could be observed between polysorbates, while tyloxapol yielded a bigger area (indicated in grey in Figure 2) of macroscopically transparent formulations, with a higher number of possible NLC template candidates. Optically transparent formulations could be observed at water percentages over 50% in the case of both polysorbates only when the surfactant-to-lipid percentage was higher than 80%. Tyloxapol was able to produce transparent formulations at only 60% of surfactant (with respect to the surfactant–lipid mixture) indicating enhanced stabilization capabilities. Tyloxapol is a non-ionic oligomeric surfactant with an ultra-low micellization point (0.018 mM), in comparison to its corresponding monomeric surfactant (Triton X-100, CMC: 0.22 mM), in combination with higher surface tension lowering abilities, which could be beneficial to produce less-toxic NLC [52,53]. In fact, it is also present in several available formulations due to its good ocular tolerability [54,55].

**Figure 3:**
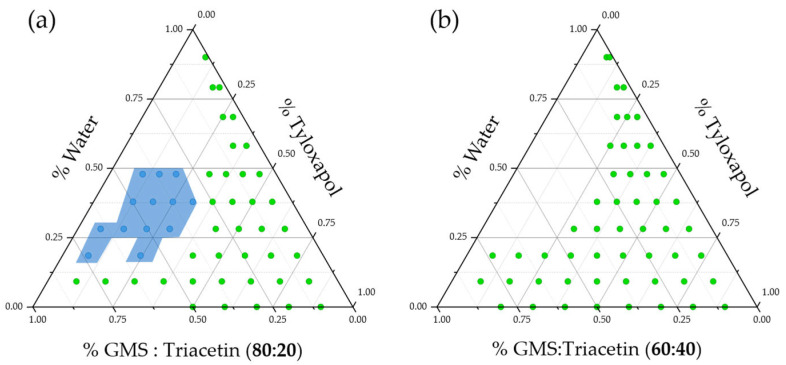
Pseudoternary phase diagrams for the water/tyloxapol/lipid mix pseudoternary systems when lipid mix was composed of (**a**) 80% GMS:20% triacetin and (**b**) 60% GMS:40% triacetin. Note how the large gel-like region (light blue) in (**a**) disappeared once GMS content was reduced (**b**).

During the titration studies, a relatively large region of candidate points appeared as gel-like structures when only 20% triacetin was present in the lipid mixture. As previously stated, GMS is an amphiphilic lipid, also used as surfactant. A simple water–GMS mixture exerts a plethora of lyotropic mesomorphs depending on the relative proportions and the working temperature. In the current work, we hypothesized that the duality of the water–GMS mixture behavior could be helpful to further reduce the surfactant content in the formulation, increasing ocular tolerability. Nonetheless, with the aim of reducing the gel-like region, we performed the same titration for the 60% GMS: 40% triacetin lipid mixture. The results are presented in Figure 3 and demonstrate that reducing the GMS content in the formulation leads to elimination of the gel-like region and to an increase in the number of microemulsion templates.

The undesired gel-like phases usually occurred at low tyloxapol concentration, where GMS was the predominant molecule, and thus might be acting as jellifying agent [52,56]. In addition, optically transparent microemulsions were spontaneously obtained without cosurfactant addition.

### 3.4. Hot Stage Polarized Light Microscopy

**Figure 4:**
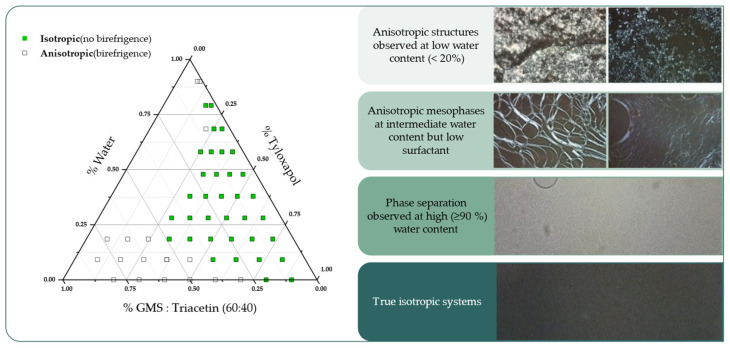
Pseudoternary phase diagram corrected after microscopic characterization under polarized light (left) and different micrographs of the observed anisotropic structures (right).

Macroscopical transparency is not an absolute indicator of isotropy, which is a fundamental characteristic of a microemulsion [57]. In this sense, we observed the macroscopically transparent candidates under polarized light in order to identify the isotropy/anisotropy of the systems for the pseudoternary system (60% GMS: 40% triacetin)/tyloxapol/water. The resulting pseudoternary phase diagram summarizing the findings is depicted in Figure 4, in addition to photomicrographs of the observed mesophases.

At low water concentrations, highly birefringent spherulitic-like structures were observed which could be mainly explained due to the presence of unmelted GMS alpha-crystals [58,59]. Above 20% water content, isotropic systems can already be observed at high surfactant concentrations, while with low surfactant concentrations birefringent liquid crystalline mesophases appear. No efforts were made on further characterization of these birefringent mesophases since the focus of interest is isotropic systems which appear dark under polarized light. Unexpectedly, some formulations showed a clear phase separation at high water concentrations (≥90% water content), which had not been identified previously.

The developed method reduced the initial 53 candidate points to only 37 possible formulations. Nonetheless, among the both isotropic and transparent candidates, different colloidal systems can still exist such as bicontinuous, oil-in-water and water-in-oil microemulsions. Therefore, a final step based on trial and error is required for the NLC production.

### 3.5. NLC Preparation and Characterization

Three different regions of the proposed pseudoternary phase diagram were studied to produce NLC using warm microemulsion templates. Particularly, (1) a low water microemulsions region (below 50% water), (2) a rich water region (with 90% water in the warm microemulsion), and (3) the previously obtained region of isotropic, optically transparent candidates. Percentage of drug was fixed at 4% with respect to the mass of lipid mix present in each formulation. Table 1 summarizes the main findings.

As expected, redispersion 1:20 in cold water of the templates with less than 50% water (L1, L2, Table 1) led to instant solidification of the redispersing drop, indicating that the external phase of the microemulsion was the lipid. Therefore, isotropy of the system was not enough to predict their suitability.

**Figure 5:**
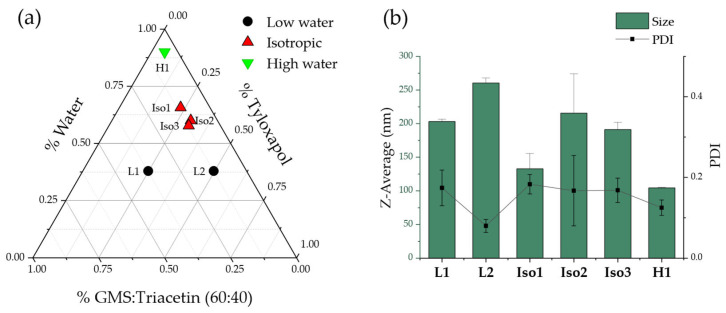
Pseudoternary phase diagram (**a**) with the composition of the six studied formulation candidates and (**b**) size and PDI values of the obtained NLC at t = 24 h after production. Data values are mean ± standard deviation (*n* = 3).

Three different candidates from the isotropic and transparent region led to adequate DLS results, compatible with the ocular route (<200 nm Z-average, desirable PDI of 0.1) confirming initially the adequacy of our hypothesis (Figure 5).

A third region was explored, with high (90%) water content in the warm template, which also led to satisfactory results. In fact, high water content microemulsions are preferred for the selected method since they allow for better pipetting and thus less drug loss in the preparation process. Small changes in the composition between Iso1, Iso2 and Iso 3 led to relatively large changes in the nanoparticle size, but on the other hand, an important increase in the water content did not produce any significant changes in the obtained nanoparticles.

Based on the collected results, formulation H1, with a composition of 75 mg GMS, 50 mg triacetin, 125 mg tyloxapol, and 2.25 mL water as the warm microemulsion template, was selected. The warm microemulsion was then redispersed 1:20 in cold water for globule solidification and NLC production. The resulting NLC presented a Z-average of 104.4 ± 0.8 nm, PDI of 0.125 ± 0.019 and zeta potential of −6.51 ± 0.575 mV. Drug loading of the formulation was quantified only after tangential flow filtration of the NLC, which also allowed concentration of the NLC previously diluted in the 1:20 dispersion step. The final drug concentration was approx. 0.2 mg/mL for dexamethasone acetate (DexAc-NLC) and approx. 0.15 mg/mL for dexamethasone (Dex-NLC).

#### NR Loaded NLC

NR is a hydrophobic heterotetracyclic fluorescent probe, which is used in bioimaging due to its high affinity for nonpolar media [60]. In addition, NR is a solvatochromic dye, i.e., its absorption and emission spectra are sensitive to the polarity of the surroundings, making it a useful fluorescent probe for different local environments [61,62].

Blank NLC are fluorescent in the UV region, as reported in Figure S3. This weak fluorescence is attributed to the tyloxapol surfactant, which has the same emission profile. However, the emission of tyloxapol did not affect MPM experiments, since it falls in a spectral region that cannot be detected with the applied MPM setup.

The produced NR-NLC was purified from unencapsulated Nile red via size exclusion chromatography, with the help of desalting columns from which several fractions were collected (Table S1). No differences have been observed between the emission (and excitation) spectra of the unseparated sample and of the filtered fractions, suggesting that NR is probably embedded in a similar environment (Figure S4).

**Figure 6:**
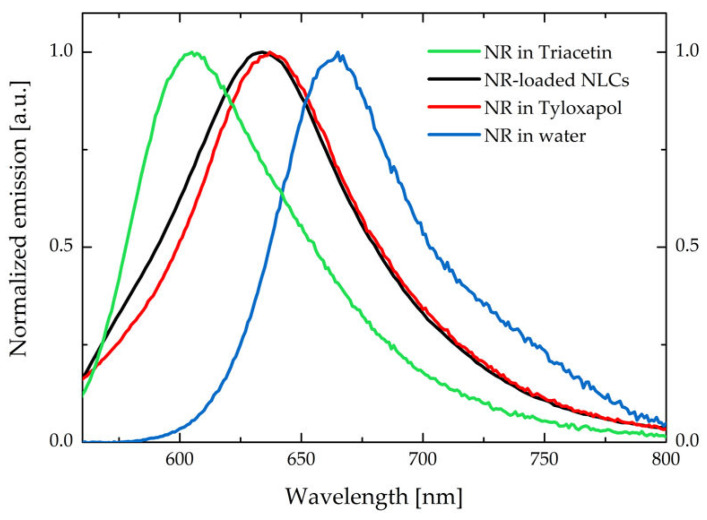
Comparison between emission spectra of NR in triacetin, NLC (aqueous suspension), tyloxapol (2.7 mg/mL in water), and water (with <1% DMSO). The spectra have been collected exciting at 500 nm.

The successful encapsulation of NR inside the NLC is evident from the comparison between emission spectra in different media reported in Figure 6 and the MPM images reported in Figure S5.

The emission spectrum of the NR-NLC is very similar to the one collected from NR at a concentration of 1 µM in a ~2.7 mg/mL tyloxapol aqueous solution (corresponding to tyloxapol concentration in the NLC formulation). Emission spectra suggest that NR is entrapped between the polyoxyethylene chains of the surfactant, i.e., in the surface layer of the NLC. The similarity of the emission spectra of NR collected in tyloxapol and NLC does not exclude the presence of some NR-loaded tyloxapol micelles in the suspension, which could be in thermodynamic equilibrium with the NLC and thus, not completely removed by the size exclusion chromatography. This assumption is further supported by the presence of a non-negligible background emission signal in the MPM images reported in Figure S5, that could stem from the presence of NR-loaded tyloxapol micelles.

### 3.6. Validation of a DexAc Extraction Method from Porcine Sclera and Choroid

**Figure 7:**
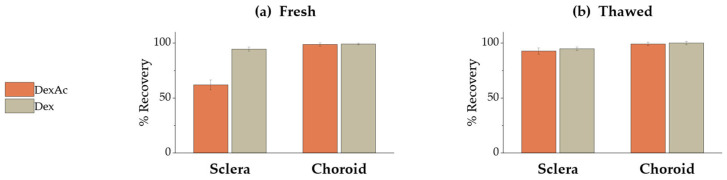
Percentage recovery of DexAc (orange) and Dex (grey) from fresh (**a**) and thawed (**b**) isolated sclera and choroid. Data were collected by applying either DexAc or Dex to ocular tissues.

A method for extracting both DexAc and Dex from porcine sclera and choroid was developed and validated. The extracting procedure consisted of soaking fresh tissue samples in an acetonitrile/water mixture (65:35, *v*/*v*) for 2 h at room temperature. While both DexAc and Dex were successfully extracted from choroid, only Dex recovery from sclera was satisfactory (i.e., >90%), as shown in Figure 7a.

The amount of DexAc retrieved from scleral samples was approx. 60%, while a significant amount of Dex was detected in the sample (data not shown). DexAc lowering in favor of Dex formation could be attributed to an enzymatic bioconversion boosted by esterases, which are ubiquitous enzymes expressed in mammal eyes [63]. Particularly, among the porcine ocular tissues, sclera shows the higher hydrolysis rate [64]. To verify this hypothesis, the experiment was repeated using frozen and thawed tissues, considering that the freezing process could decrease the esterase activity. As shown in Figure 7b, the amount of DexAc was significantly higher compared to fresh sclera (92.8 ± 2.7% vs 61.9 ± 4.6%, respectively) confirming the metabolism. On the contrary, despite esterase expression within choroid, DexAc was not metabolized under the conditions tested. In any case, Dex recovery was always above 90%.

### 3.7. Ex Vivo Ocular Tissues: Permeation and Retention Experiments

Ex vivo permeation and retention experiments were performed using both freshly excised porcine sclera (referred to as S) and sclera with choroid (SCh). With the aim of increasing dexamethasone acetate concentration, DexAc-NLC was subjected to tangential flow filtration, thus reaching the final concentration of approx. 0.2 mg/mL. No dexamethasone was detectable in the formulation. Since, as demonstrated above, dexamethasone acetate undergoes metabolism when in contact with fresh ocular tissues, and dexamethasone is produced, data are presented as total amount (nmol) of both DexAc and Dex.

No detectable concentrations of DexAc or Dex could be observed after 24 h when a reference oily solution of DexAc in triacetin (250 μg/mL corresponding to 575.4 μM) was used as donor for permeation experiments across sclera and sclera-choroid. These results can be explained by the partition coefficient: DexAc finds a favorable environment within the hydrophobic triacetin, thus the transscleral diffusion does not take place.

Permeability coefficients were calculated from permeation profiles reported in Figure S6a, applying Equation 1 to the linear portion, defined by the three last timepoints. Considering the sum of DexAc and Dex, *P_app_* was 1.7 ± 0.6 × 10^−6^ cm/s for sclera and 1.2 ± 0.4 × 10^−6^ cm/s for sclera-choroid. After 24 h of permeation experiment, no statistical differences were detected (*p* > 0.05): choroid presence apparently did not hinder the diffusion of both compounds, as also demonstrated by the lag-time, which was calculated considering DexAc+Dex, and resulted approx. 11 h in both cases. Permeability coefficients for DexAc across sclera, to the best of our knowledge, are not available in the literature. However, the *P_app_* for Dex referred to porcine sclera is 11.1 ± 2.1 × 10^−6^ cm/s, one order of magnitude higher than the value observed in our experience [65]. This difference can be easily explained considering the time required for the release of DexAc from NLC, as well as for Dex formation via DexAc metabolism. In fact, when a Dex-NLC formulation was applied to SCh (Figure S6b), the obtained *P_app_* value was 1.6 ± 0.1 × 10^−6^ cm/s, while the lag time was approx. 3.5 h. Slight differences in *p* values might be attributed to different pig breeds affecting the biologic variability, as previously reported in [66].

Our findings confirm a relevant role of esterase on DexAc, even on isolated ex vivo tissues, as already shown in Figure 7. At the end of the experiment, when studying the permeation across the sclera, the amount of the prodrug was 2.9 times lower than for the Dex, which is formed by enzymatic process (10.1 ± 3.2 nmol/cm^2^ vs 29.0 ± 7.6 nmol/cm^2^). The underpinning mechanism that might justify this result is that the ester prodrug, due to its high logP of 2.65, will rather be retained inside the intact NLC than diffuse into the highly hydrophilic sclera. Nonetheless, the released DexAc is quickly converted into dexamethasone, a less lipophilic compound (logP = 1.9), which diffuses more easily across the sclera. At the same time, when the double barrier sclera-choroid was present, still a difference was observed, being the amount permeated 5.4 ± 1.8 nmol/cm^2^ for DexAc and 23.5 ± 6.9 nmol/cm^2^ for Dex, respectively. This outcome confirms that, by selecting the acetate ester prodrug, a sustained Dex release was achieved (in comparison to Dex-NLC) since the limiting step in the process is the enzymatic conversion of the ester prodrug into dexamethasone.

In addition to permeation, retention plays a relevant role in view of a periocular administration. In fact, it has been previously demonstrated for compounds having different physicochemical properties that sclera acts as a drug reservoir, allowing for a sustain release over time and ultimately for a reduced frequency of administration [67,68].

Table 2 reports the retention data collected after 24 h of contact between DexAc-NLC and Dex-NLC and ocular tissues (further data are available in Table S2).

While Dex was retained in both sclera and choroid, DexAc was retrieved essentially from sclera. Since the NLC present an excessively large size to permeate intact through the sclera, and DexAc has a high LogP, it cannot be ruled out that further structures, such as tyloxapol micelles, might be instantly formed with the degradation products of the NLC. This hypothesis has been further assessed in the next section.

### 3.8. Tissue Distribution of NR through MPM Studies

**Figure 8:**
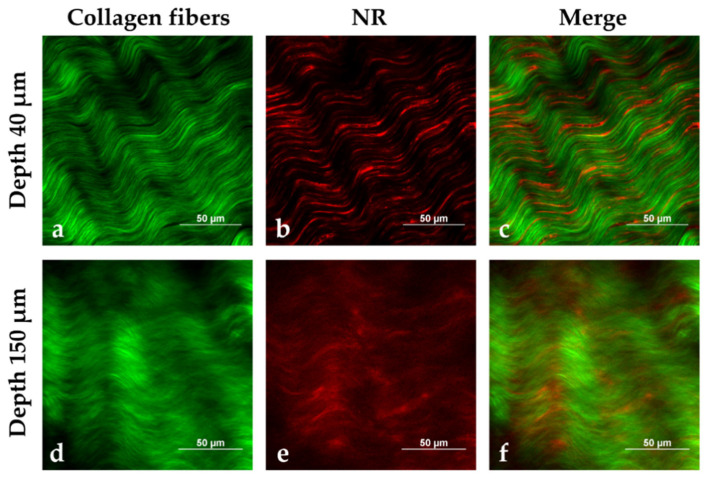
MPM images of porcine sclera (excitation wavelength: 1100 nm) after 2 h of contact with NR-loaded NLC (0.1 μg/mg triacetin), at a depth of 40 μm (**a**,**b,c**) and 150 μm (**d**,**e**,**f**) from the tissue surface, respectively: (**a** and **d**) Signal detected in the green channel, mainly due to the SHG of collagen fibers; (**b** and **e**) Signal collected in the red channel, which is attributed to NR emission; (**c** and **f**) Corresponding channels overlay, showing NR distribution (red) between collagen fibers (green). Images size: 170 μm × 170 μm.

To further investigate the behavior of the NLC once in contact with porcine sclera, NR distribution from NR-NLC was studied by multiphoton microscopy, following the same experimental approach that we recently proposed [69]. MPM is a non-invasive fluorescence imaging technique that allows creation of tridimensional images of isolated ex vivo tissues. In-depth images can be obtained by interpretation of diverse signals such as the two-photon excited fluorescence (TPEF) of fluorophores, or more complex nonlinear signals inherent to the tissue, such as the second harmonic generation (SHG) [70]. Figure 8 reports images acquired at 40 and 150 μm depth from the episclera (the outer face of the sclera).

**Figure 9:**
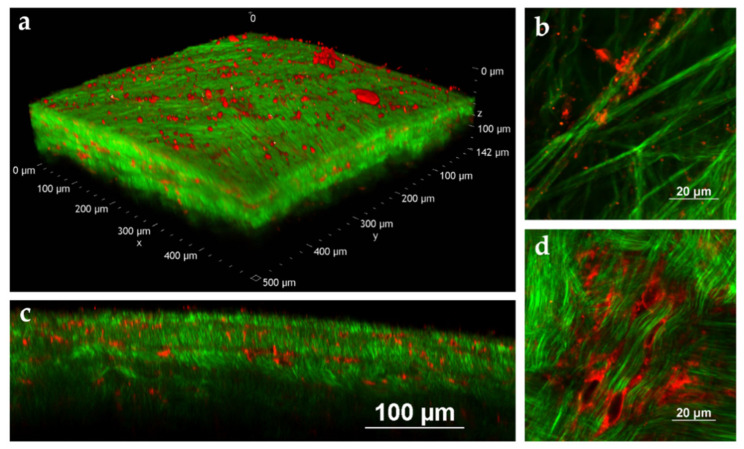
MPM images of porcine sclera after 2 h contact with NR-loaded NLC (0.1 μg/mg triacetin). (**a**) Volume rendering of the tissue reconstructed from the Z-stack (Z-step: 1 µm, total depth: 142 µm), (**b**) image of the tissue surface (size: 82 μm × 82 μm), (**c**) YZ slice extracted from the Z-stack reported in panel a (image size: 512 μm × 142 μm), (**d**) image acquired 30 μm below the surface of the tissue (size: 92 μm × 92 μm). The excitation wavelength was set to 1100 nm for all the collected images.

The green signal is mainly attributed to the SHG of collagen fibers; the black spaces among green fibers are aqueous pores (Figure 8a,d), since sclera is composed of 70% water [71]. Collagen fibers appear thicker in the outermost scleral layers (Figure 8a) than in the innermost ones (Figure 8d), as previously observed with a different technique [72]. NR emission, collected in the red channel (Figure 8b,e), was detected between the collagen fibers, as clearly observed in panels c and f of Figure 8. Figure 9 reports both a volume rendering and bidimensional images of the permeated sample with NR-loaded NLC. On the surface of the tissue (Figure 9 panels a and b), some fluorescent particles and big aggregates were present, adhered to the most externals collagen fibers.

The emission signal of NR was detected from scleral porosities up to the innermost tissue layers that have been visualized, 142 μm from the surface.

NR is a hydrophobic probe characterized by very weak emission in aqueous environments, primarily due to aggregation phenomena [73,74]. The bright emission observed in the red channel suggests that NR is well solubilized even after the permeation in the tissue.

**Figure 10:**
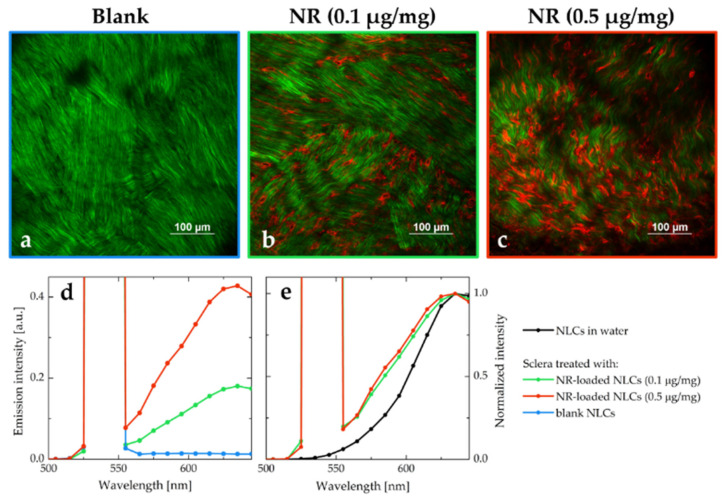
MPM images of porcine sclera (depth of 40 μm, image size: 512 μm × 512 μm and excitation wavelength of 1100 nm) after 2 h of contact with NLC: (**a**) blank NLC, (**b**) NR-loaded NLC (starting from 0.1 µg/mg NR), (**c**) NR-loaded NLC (starting from 0.5 µg/mg NR). Panels (**d** and **e**) report the TPEF (broad band)/SHG (intense sharp peak; peak maximum is not shown) profiles obtained with the spectral detector in correspondence of images (**a**,**b**,**c**) focal planes, exciting at 1080 nm. The spectra in panel e have been normalized and compared to the emission signal collected with the microscope from a NR-loaded NLC aqueous suspension. Images were acquired in the same experimental conditions (detector gains and laser power), as well as emission spectra.

The emission spectra of the tissue after the permeation of NLC both loaded with NR (starting from two different concentrations of NR in triacetin, namely 0.1 μg/mg and 0.5 μg/mg) and without dye were also recorded. The three emission profiles obtained are reported in Figure 10, together with the MPM images collected in correspondence of the same focal planes (a comparison between the corresponding Z-stacks is reported in Figure S7).

All the emission profiles were characterized by a strong and sharp peak centered at 540 nm, due to the SHG process promoted by the collagen fibers. The spectrum collected from the tissue permeated with blank NLC (blue line in Figure 10d) has a very intense SHG signal, and very weak autofluorescence. The spectra obtained from the tissues treated with NR-NLC were broadened when compared to the emission recorded from NR-NLC in aqueous suspension (Figure 10e). The broadening of spectra is often associated with an increase of the disorder in the local environment [75]. As hypothesized in Section 3.7, it is likely that the NLC do not permeate intact within the tissue: in this case, a redistribution of the NR molecules would be expected, exposing the dye to the lipophilic components of the nanocarriers, as well as to the hydrophilic surfactant and the scleral fibroblasts. The resulting emission spectrum is characterized by an overall broadening since it originates from the superposition of emission spectra of NR in different environments.

## 4. Conclusions

We reported the development, characterization and optimization of DexAc-loaded NLC intended for periocular administration. NLC was prepared with an easy and potentially scalable method which did not involve the use of organic solvents or co-surfactants. All the excipients used are compatible with ophthalmic administration.

Ex vivo studies carried out with porcine ocular tissues, also supported by the evidence collected via multiphoton microscopy, demonstrated retention of the DexAc within scleral tissue following application of NLC. Thanks to the ocular enzymes, Dex is progressively developed, allowing for a sustained release over time of the active metabolite. The collected results are encouraging and suggest the possibility of development on a larger scale. Further investigations are needed for a deeper comprehension of these systems and to approach relevant issues such as sterilization and biocompatibility.

## Figures and Tables

**Table 1:** Composition of six selected microemulsion templates, their macroscopic and microscopic appearance and the macroscopic result obtained after 1:20 redispersion in cold water. Note how even if a formulation is both transparent and isotropic it can still lead to inadequate redispersion products (L2) and less macroscopically transparent microemulsions can still lead to adequate NLC production (H1). All formulations were loaded at 4% DexAc with respect to their lipid mix content.

Phase Diagram Region	Code	Lipid Mix % (GMS: Triacetin 60:40)	Surfactant % (Tyloxapol)	Water %	Warm Microemulsion		Redispersed 1:20 Product
					Transparent	Isotropic	
Low water	L1	37.3	24.8	37.9	Y	N	>1 mm pellets
	L2	12.4	49.7	37.9	Y	Y	>1 mm pellets
Isotropic	Iso1	11.1	23.2	65.7	Y	Y	Slightly bluish, milky
	Iso2	10.1	29.9	60	Y	Y	Slightly bluish, milky
	Iso3	12	30.3	57.7	Y	Y	Slightly bluish, milky
High water	H1	5	5	90	N	Y	Slightly bluish, milky

**Table 2:** Retention within fresh porcine tissues after 24 h contact at 37 °C in Franz cells with DexAc-NLC and Dex-NLC (S = sclera; Ch = choroid; SCh = sclera + choroid).

		DexAc-NLC (230 μg/mL = 529.3 μM) (*n* = 8)	Dex-NLC (141 μg/mL = 359.9 μM) (*n* = 4)
Sample	Tissue	DexAc+Dex (nmol/g tissue)	Dex (nmol/g tissue)
S	S	139.9 ± 35.6	Not studied
	S	98.9 ± 19.9	83.4 ± 8.1
SCh	Ch	30.9 ± 9.3	80.7 ± 12.6
	SCh	92.8 ± 19.0	83.3 ± 8.3

## Data Availability

Raw data are available upon request.

## Supplementary Materials

The following are available online at https://www.mdpi.com/article/10.3390/pharmaceutics15020407/s1, Figure S1: Melting thermograms (from 25 to 90 °C) and polymorphic events recorded 5 minutes, 1 day, 1 week and 1 month after solidification of different lipid melts containing Imwitor® 491 (GMS) with increasing concentrations of liquid triacetin (top) and numeric values for the observed temperature peaks (bottom). Pure GMS is described by the discontinuous line. Figure S2: Melting thermograms (from 25 to 90 °C) and polymorphic events recorded 5 minutes, 1 day, 1 week and 1 month after solidification of different lipid melts containing Imwitor® 491 (GMS) with increasing concentrations of liquid triacetin. Pure GMS as a discontinuous line. Figure S3: Normalized absorption, excitation, and emission spectra of a diluted plain NLC aqueous suspension. The excitation and emission wavelengths are reported in the legend. Figure S4: Comparison between the excitation and emission spectra of the unseparated sample and the two separated fractions of NR-loaded NLC. The emission and excitation spectra have been acquired using appropriate longpass filters with 550 and 645 nm cut-off wavelengths, respectively. Figure S5: MPM images obtained from NR-loaded NLC with an excitation wavelength of 1100 nm. Images size: 512 μm × 512 μm (left) and 101 μm × 94 μm (right, white line length: 5 μm). Figure S6: (a) Dex+DexAc permeation profile across S and SCh from DexNLC (donor concentration 230 μg/ml, corresponding to 529.3 μM DexAc). (b) Dex permeation profile across SCh from DexNLC (donor concentration 204 μg/ml, corresponding to 519.8 μM Dex). Figure S7: Volume renderings reconstructed from the Z-stacks of the scleral tissues treated with blank NLC (Z-step: 1 µm, total depth: 142 µm, panel a and b), 0.1 µg/mg NR-loaded NLC (Z-step: 1 µm, total depth: 142 µm, panel c and d), 0.5 µg/mg NR-loaded NLC (Z-step: 1 µm, total depth: 192 µm, panel e and f). Panels a, c and e report the volume overviews while in panels b, d and f the XY views can be observed. The same experimental conditions were used to acquire the three Z-stacks: laser power, detector gain and excitation wavelength (1100 nm). Table S1: DLS characterization of the eight fractions collected after separation in desalting column. Table S2: Retention within fresh porcine tissues after 24 h contact at 37 °C in Franz cells with DexAc-NLC (230 μg/mL = 529.3 μM). (S = sclera; Ch = choroid; SCh = sclera + choroid). References [39,43,44,45,46,47,48] are also cited in the Supplementary Materials.
