# Supplementary material for: Nanostructured Lipid Carriers for Enhanced Transscleral Delivery of Dexamethasone Acetate: Development, Ex Vivo Characterization and Multiphoton Microscopy Studies
Source: Pharmaceutics. 2023 Jan 25;15(2):407. doi: 10.3390/pharmaceutics15020407 (PMC9961953; doi:10.3390/pharmaceutics15020407)
Supplement: Supplementary file 1 [file pharmaceutics-15-00407-s001.zip › pharmaceutics-2106234-supplementary.pdf]

pharmaceutics

Supplementary Materials: Nanostructured Lipid Carriers for Enhanced Transscleral Delivery of Dexamethasone Acetate: Development, Ex Vivo Characterization and Multiphoton Microscopy Studies

Felipe M González-Fernández, Andrea Delledonne, Sara Nicoli, Paolo Gasco, Cristina Padula, Patrizia Santi, Cristina Sissa and Silvia Pescina

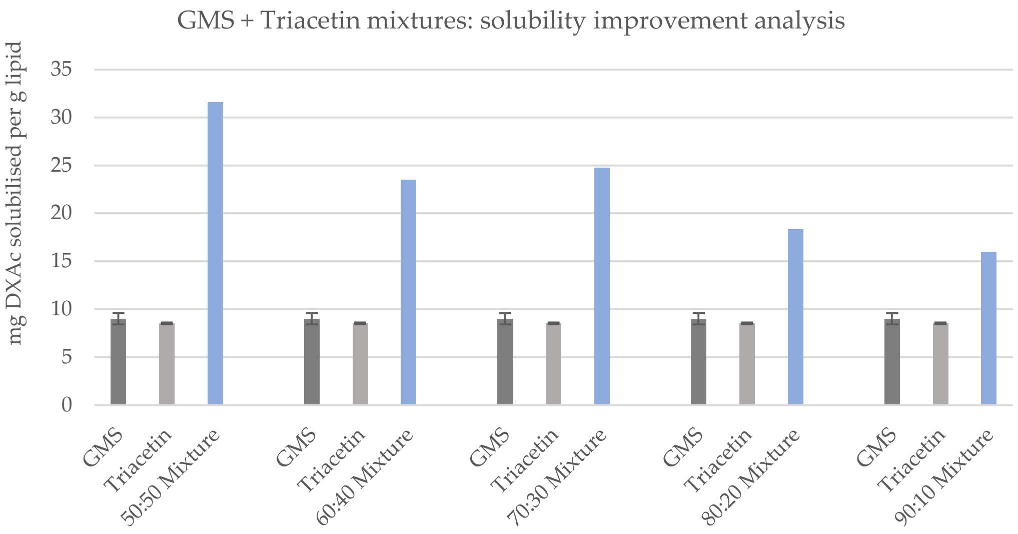

Figure S1. Solubility of dexamethasone acetate (five degrees over the melting temperature, 78 °C in case of Imwitor® 491) in different mixture ratios of Imwitor® 491 (GMS) and triacetin.

Supplementary data: Liquid-solid lipid mixture characterization by Differential Scanning Calorimetry

Solid lipids are known to recrystallize in different polymorphs upon melting, since the apolar chains can pack themselves in different arrangements. The number of polymorphic species to be observed is related, amongst others, to the lipid nature and the time since solidification. Generally, quick solidification of a melt will lead to amorphous, less stable polymorphs, where the long hydrophobic chains are poorly arranged. Over the time, polymorphic transitions towards a more thermodynamically stable packing are observed, minimizing the free Gibbs energy [39].

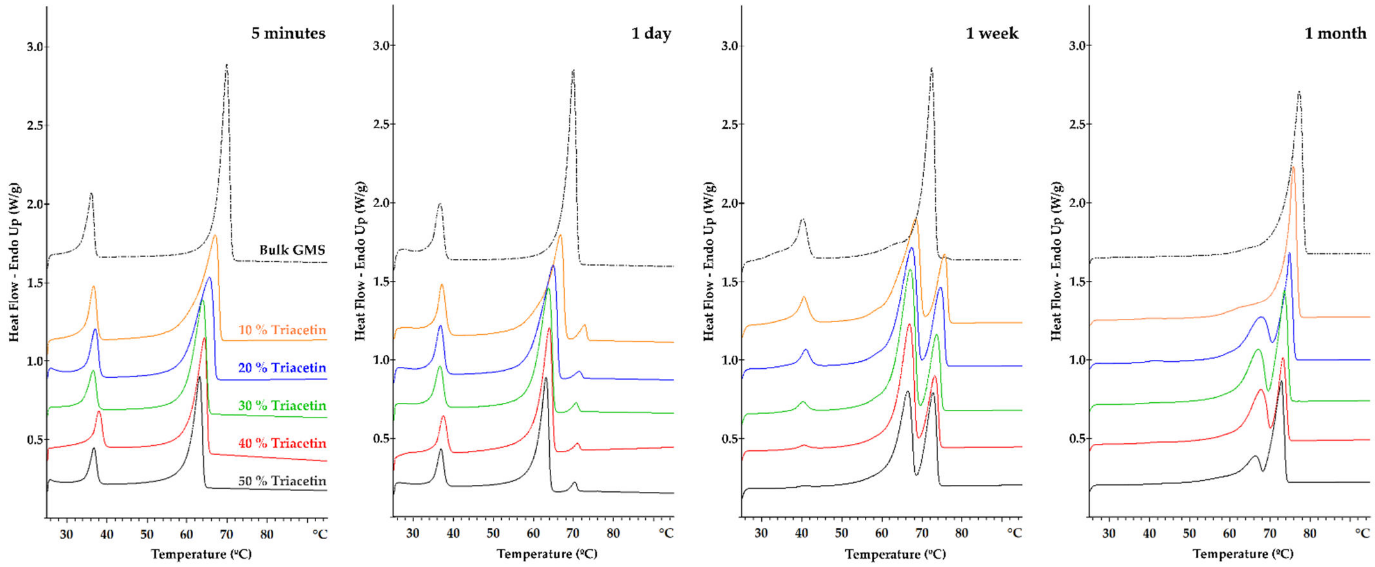

	5 min			1 day			1 week			1 month		
	Peak 1	Peak 2	Peak 3	Peak 1	Peak 2	Peak 3	Peak 1	Peak 2	Peak 3	Peak 1	Peak 2	Peak 3
Bulk GMS	36.5	70.5	*	37.4	70.6	*	41.2	73.1	*	*	*	78
10% Triacetin	37.2	68	*	37.9	67.5	73.1	41	69.5	76.3	*	*	76.5
20% Triacetin	37.8	67	*	37.6	66	72	41.7	68.8	75.5	*	69.7	75.7
30% Triacetin	37.2	64.9	*	37.4	64.7	70.2	41	68.4	74.6	*	68.6	74.5
40% Triacetin	38.7	64.9	*	38.3	64.6	71.5	41	68	74	*	69.3	74.1
50% Triacetin	37.2	63.8	*	37.5	63.8	70.9	*	67.6	73.7	*	67.4	73.6

Reported values correspond to peak temperatures (T_m) in °C.

*No peak was detected

Figure S2. Melting thermograms (from 25 to 90 °C) and polymorphic events recorded 5 minutes, 1 day, 1 week and 1 month after solidification of different lipid melts containing Imwitor® 491 (GMS) with increasing concentrations of liquid triacetin (top) and numeric values for the observed temperature peaks (bottom). Pure GMS is described by the discontinuous line. The resolidified pure monoglyceride shows two endothermic melting peaks at 36.5 °C and 70.5 °C during the first hours after re-solidification. The low temperature melting peak (i.e., Peak 1 at 1 day) is believed to correspond to a sub-alpha-form, which is not present after one month storage at room temperature. Previous studies indicate presence of this sub-alpha form until 36 days after solidification but, in our case, it could not be detected already after 30 days. The second peak at 70.5 °C (initial hours) represents the alpha-form, as confirmed by XRD studies elsewhere [43], where it has been reported up to 16 days after solidification. After one month, all the GMS has evolved to a polymorph with a melting peak at 78 °C which might correspond to the more stable beta triclinic cell polymorph, as confirmed in previous XRD studies, where this beta-form starts to be detectable 24 days after solidification of the melt [44-46]. Partial substitution of the bulk GMS with triacetin (≥10% ratio) leads to an intermediate peak (denoted as Peak 2) which progressively decreases but is still detectable after one month. The peak temperature value coincides with that of the alpha-form observed in the initial hours for the bulk GMS, but it appears downshifted by the increasing triacetin concentration. Triacetin (already at 10%) appears to interact with the functional groups of GMS, hindering the tendency of bulk GMS to rearrange into more densely packed lattices (i.e. single peak at 78 ºC after 1 month in the bulk GMS). Addition of 10% triacetin is nonetheless insufficient to maintain this effect after 1 month, while the concentrations of ≥ 20% triacetin still maintain this intermediate peak. On the other hand, if data at 1 week is observed, it appears that increasing triacetin concentrations promote conversely the elimination of the sub-alpha form (approx. 41 ºC), an effect also previously reported with similar oil-lipid mixtures [47,48].

Table S1. DLS characterization of the eight fractions collected after separation in desalting column.

Collected 1mL Fraction	Z-Average Size (nm)	PDI
Unfiltered	106.9 ± 1.2	0.104 ± 0.019
Fraction 1	1042 ± 282.1	0.916 ± 0.118
Fraction 2	669 ± 213.5	0.67 ± 0.16
Fraction 3	109.3 ± 1.5	0.126 ± 0.007
Fraction 4	108.2 ± 1.8	0.114 ± 0.007
Fraction 5	110.5 ± 1.4	0.133 ± 0.008
Fraction 6	118.8 ± 3.1	0.207 ± 0.064
Fraction 7	367.8 ± 124.2	0.412 ± 0.126
Fraction 8	354.0 ± 126.9	0.401 ± 0.076

Data reported are mean ± standard deviation (n = 3)

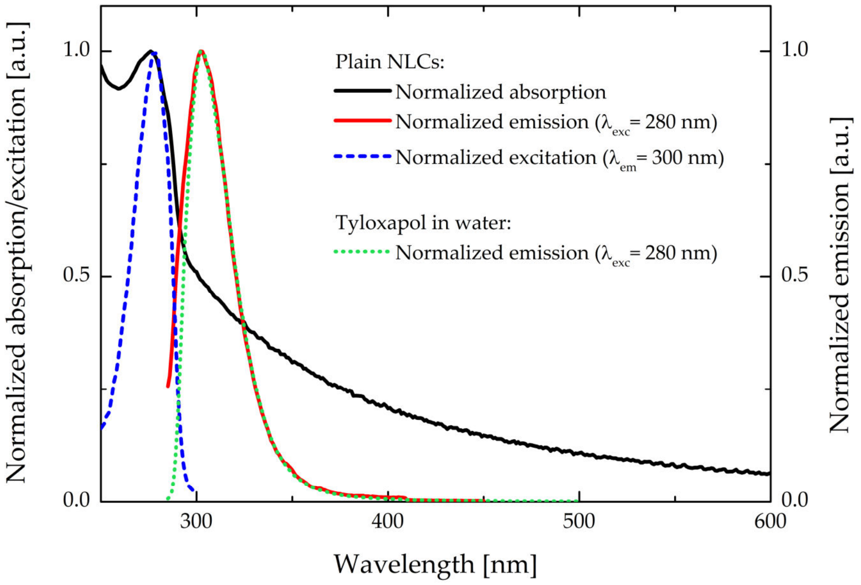

Figure S3. Normalized absorption, excitation, and emission spectra of a diluted plain NLC aqueous suspension. The excitation and emission wavelengths are reported in the legend.

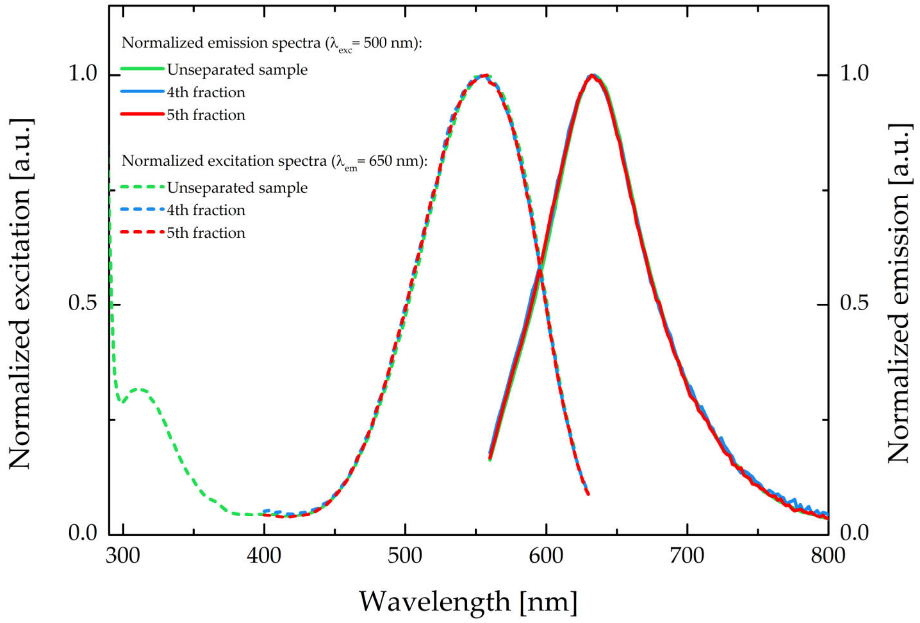

Figure S4. Comparison between the excitation and emission spectra of the unseparated sample and the two separated fractions of NR-loaded NLC. The emission and excitation spectra have been acquired using appropriate longpass filters with 550 and 645 nm cut-off wavelengths, respectively.

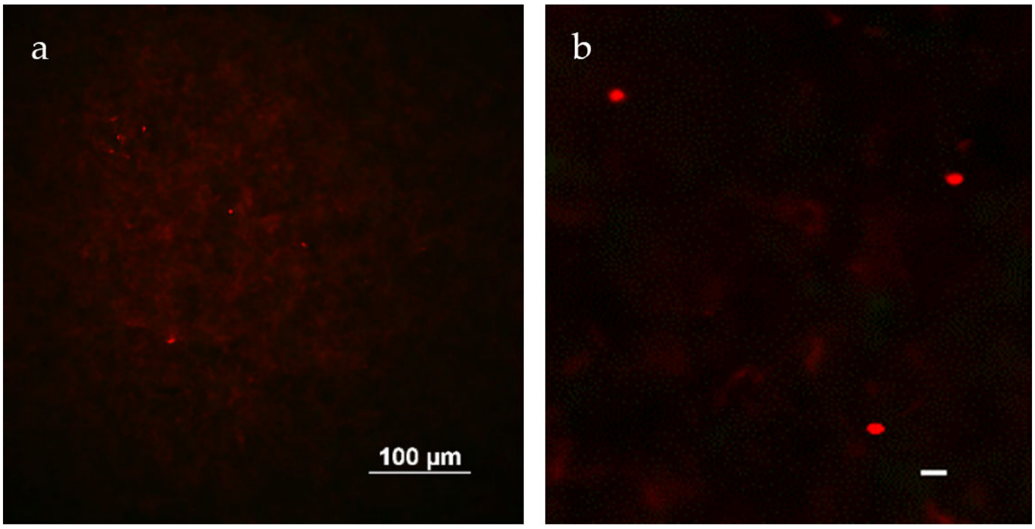

Figure S5. MPM images obtained from NR-loaded NLC with an excitation wavelength of 1100 nm. Images size: 512 µm x 512 µm (a) and 101 µm x 94 µm (b, white bar length: 5 µm).

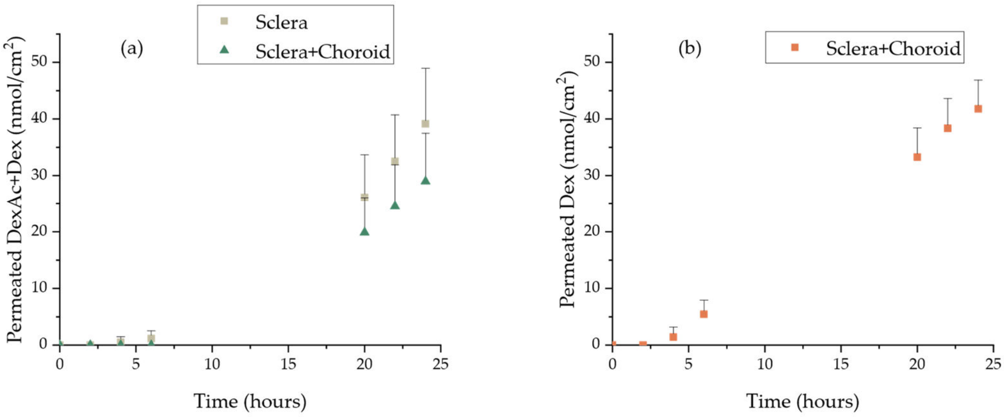

Figure S6. (a) Dex+DexAc permeation profile across S and SCh from DexAc-NLC (donor concentration 230 µg/mL, corresponding to 529.3 µM DexAc). (b) Dex permeation profile across SCh from Dex-NLC (donor concentration 141 µg/mL, corresponding to 359.9 µM Dex).

Table S2. Retention within fresh porcine tissues after 24 h contact at 37 °C in Franz cells with DexAc-NLC (230 µg/mL = 529.3 µM). (S = sclera; Ch = choroid; SCh = sclera + choroid).

**Sample**	**Tissue**	**DexAc**		**Dex**	
		(nmol)	(nmol/g tissue)	(nmol)	(nmol/g tissue)
S (*n* = 8)	S	8.9 ± 3.4	91.8 ± 34.8	4.8 ± 2.3	48.1 ± 19.5
SCh (*n* = 8)	S	4.7 ± 1.5	50.6 ± 13.4	4.6 ± 1.5	48.4 ± 13.2
	Ch	0.0 ± 0.0	0.0 ± 0.0	0.3 ± 0.1	30.9 ± 9.3
	SCh	4.7 ± 1.5	45.9 ± 12.1	4.8 ± 1.5	46.9 ± 12.7

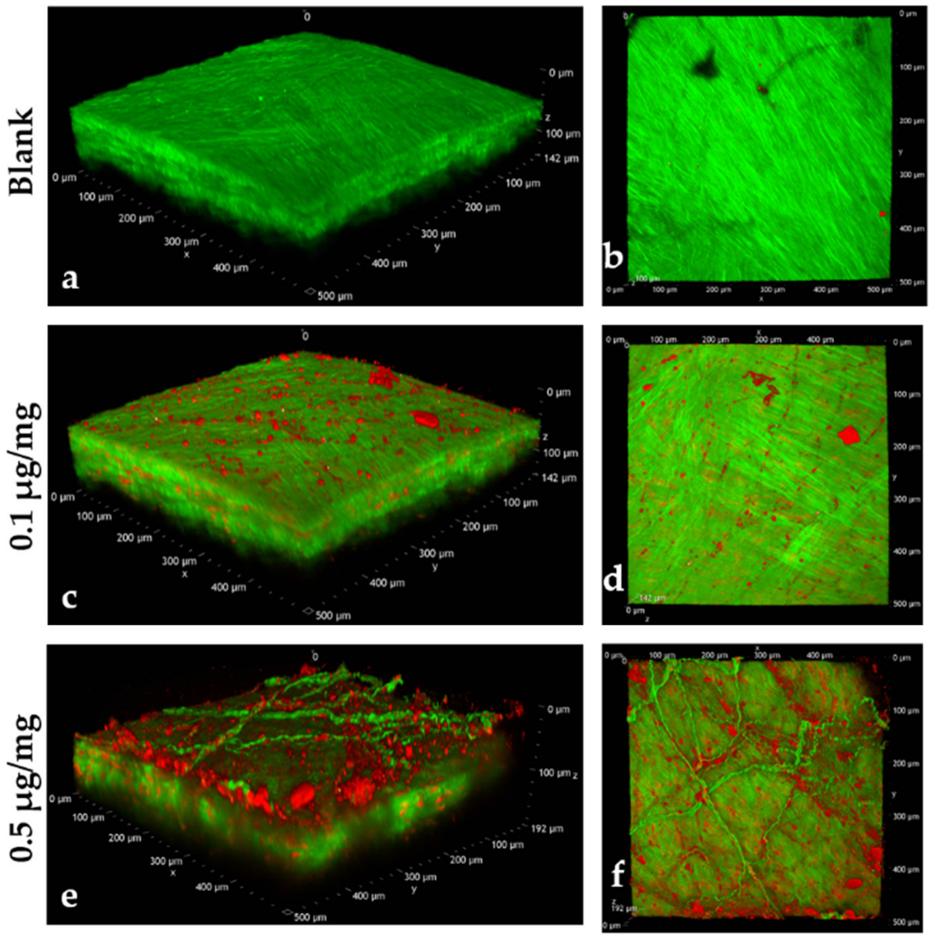

Figure S7. Volume renderings reconstructed from the Z-stacks of the scleral tissues treated with blank NLC (Z-step: 1 µm, total depth: 142 µm, panel a and b), 0.1 µg/mg triacetin NR-loaded NLC (Z-step: 1 µm, total depth: 142 µm, panel c and d), 0.5 µg/mg triacetin NR-loaded NLC (Z-step: 1 µm, total depth: 192 µm, panel e and f). Panels a, c and e report the volume overviews while in panels b, d and f the XY views can be observed. The same experimental conditions were used to acquire the three Z-stacks: laser power, detectors gain and excitation wavelength (1100 nm).
